# Bone aluminum accumulation in the current era

**DOI:** 10.1590/2175-8239-JBN-2024-0023en

**Published:** 2024-04-29

**Authors:** Rodrigo Bueno de Oliveira, Aluízio Barbosa Carvalho, Vanda Jorgetti

**Affiliations:** 1 Universidade Estadual de Campinas (Unicamp), Faculdade de Ciências Médicas, Laboratório para o Estudo Mineral e Ósseo em Nefrologia (LEMON), Campinas, SP, Brazil.; 2 Universidade Estadual de Campinas (Unicamp), Faculdade de Ciências Médicas, Departamento de Clínica Médica, Divisão de Nefrologia, Campinas, SP, Brazil.; 3 Universidade Federal de São Paulo, Escola Paulista de Medicina, Departamento de Nefrologia, São Paulo, SP, Brazil.; 4 Universidade de São Paulo, Faculdade de Medicina, Departamento de Clínica Médica, Laboratório de Fisiopatologia Renal (LIM-16), São Paulo, SP, Brazil.

**Keywords:** Chronic Kidney Disease-Mineral and Bone Disorder, Aluminum, Renal Insufficiency, Chronic, Treatment Outcome, Distúrbio Mineral e Ósseo na Doença Renal Crônica, Alumínio, Insuficiência Renal Crônica, Resultado do Tratamento

## Abstract

In the last few years, evidence from the Brazilian Registry of Bone Biopsy (REBRABO) has pointed out a high incidence of aluminum (Al) accumulation in the bones of patients with CKD under dialysis. This surprising finding does not appear to be merely a passive metal accumulation, as prospective data from REBRABO suggest that the presence of Al in bone may be independently associated with major adverse cardiovascular events. This information contrasts with the perception of epidemiologic control of this condition around the world. In this opinion paper, we discussed why the diagnosis of Al accumulation in bone is not reported in other parts of the world. We also discuss a range of possibilities to understand why bone Al accumulation still occurs, not as a classical syndrome with systemic signs of intoxication, as occurred it has in the past.

## Abstract (pt)

Nos últimos anos, evidências do Registro Brasileiro de Biópsia óssea (REBRABO) apontaram uma alta incidência de intoxicação por alumínio (Al) no tecido ósseo de pacientes com DRC em diálise. Essa surpreendente informação parece representar não apenas um acúmulo passivo deste metal, visto que dados prospectivos do REBRABO sugerem que a presença de Al no tecido ósseo pode estar independentemente relacionada a eventos cardiovasculares adversos maiores. Essas informações contrastam com a percepção mundial do controle epidemiológico dessa condição. Neste artigo de opinião, discutimos por que o diagnóstico de acúmulo ósseo de Al não é relatado em outras partes do mundo, e também discutimos uma gama de possibilidades para entender por que nós acreditamos que o acúmulo de Al no tecido ósseo ainda ocorre, não como se apresentava no passado, ou seja, como uma síndrome com sinais e sintomas sistêmicos de intoxicação.

## Introduction

About fifty years ago, nephrologists faced a devastating clinical problem: patients under hemodialysis treatment who presented dementia, anemia, and bone fractures due to mineralization disorders. The cause of this syndrome was the excess of aluminum (Al) in the blood and different organs, including the skeleton^1^. Since then, several measures have been taken to protect patients with chronic kidney disease (CKD) from Al exposure. Strategies to remove Al in the water used for hemodialysis, avoidance of Al-based phosphate binders, and drugs to increase hemodialysis clearance of Al resulted in the epidemiologic control of this condition. This control was confirmed in clinical settings, and over time, different studies documented low (apparently, safe) serum Al levels in these patients^1,2,3,4^.

However, in the last few years, contrasting evidence from the Brazilian Registry of Bone Biopsy (REBRABO) has pointed out a high incidence of Al accumulation in the bones of patients with CKD^5,6^. Clinical outcome data from this registry suggest that the presence of this metal in bone is not only an isolated histological finding. In a cohort of 275 patients with CKD followed by 3.4 years, the diagnosis of bone Al accumulation was independently associated with major adverse cardiovascular events (MACE) [HR = 3.129 (CI: 1.439–6.804; p = 0.004)]^7^.

These surprising data should make us reflect on this subject. Do these findings reflect a local problem? Do other populations in other countries also suffer from a high incidence of bone Al accumulation and may be exposed to an additional risk factor for cardiovascular complications?

Our opinion is that bone Al accumulation still occurs, but not as a classic syndrome with systemic signs of intoxication, as it has in the past. Instead, prolonged and low exposure to Al sources may result in bone Al accumulation and may be linked to adverse clinical consequences. In the next sessions, we present arguments to support our opinion.

## Why is it Plausible that Prolonged Low-Intensity Exposures to al Sources Still Occur?

The increasing incidence and prevalence of CKD places a burden on healthcare systems, particularly in terms of access to dialysis treatment. This sustained and progressive pressure in healthcare systems leads to heterogeneity in the quality of care provided and can affect the standards of water used for hemodialysis^8,9,10,11,12,13^.

**Figure 1:**
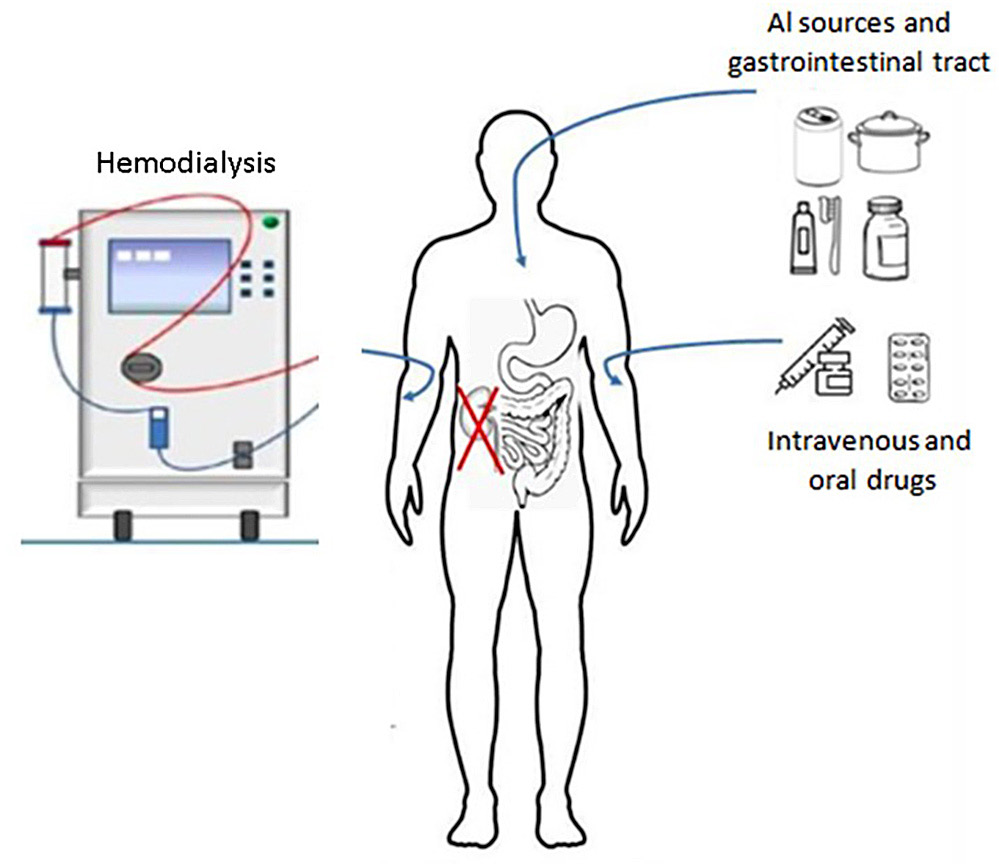
Patients with chronic kidney disease under dialysis can be exposed to many aluminum sources: water used in hemodialysis, pots and cans made of aluminum, and raw materials for oral and intravenous drugs. The current quality control for water used in hemodialysis can be inadequate in terms of frequency and maximum allowable Al limit to avoid accumulation.

Many situations have risks for exposure to Al sources, such as limited legal regulation of dialysis treatment^12^, insufficient quality control related to the presence of Al in water used for safe hemodialysis, either because of infrequency of measurements or because of inadequate cutoff levels, non-negligible amounts of Al in raw materials used in oral and intravenous drugs^14^, and Al absorption by the digestive tract^15^. Meira et al. published a thorough discussion of possible sources of Al contamination in patients in dialysis^16^. Figure 1 shows possible sources of Al exposure in patients under dialysis.

## Why is Bone al Accumulation not Reported in other Countries?

First, it is important to highlight that Brazilian public and private dialysis facilities have generally adopted the international guidelines for the prevention of Al intoxication^17^. A federal law guarantees this standard, and there is a governmental agency (*Agência Nacional de Vigilância Sanitária*) in charge of enforcing the law and carrying out preventive surveillance^18^. The reports related to Al accumulation in patients with CKD are not limited to Brazil. Other groups from China have also found Al accumulation and an association with uremic pruritus and increased mortality in hemodialysis patients^19,20^.

We propose three main possibilities to explain the actual lack of Al detection in patients with CKD under dialysis in other countries: (1) Al serum levels may not reflect Al deposition in tissue; as a cation, Al measurement may be affected by other cations such as iron and by eventual Al binding to transferrin^21^; (2) periodic measurements of serum Al levels may not reflect low and chronic exposures to Al sources^22^; (3) currently, bone Al intoxication (as a rare diagnosis) is mainly diagnosed by measurements of serum Al levels in cohorts of patients with CKD, and not by bone tissue analysis^23–33^. Table 1 summarizes the main studies on renal osteodystrophy in recent decades and its relationship with the active search for Al intoxication on bone biopsies through the gold standard technique (solochrome azurine staining). Of note, most studies did not perform (or did not report) this technique for the diagnosis of bone Al accumulation, despite performing bone biopsy and histologic analysis.

**Table 1:** Summary of studies related to incidence of bone al accumulation with study period, country, and ethnicity. Most of the studies did not provide information on this diagnosis (highlighted in black bold: na)

Author	Period	Country	Ethnicity	Sample (CKD/HD/PD)	Bone Al (%)
López *et al.* ^23^	1985–1996	BRA-URU-POR-SPA-ARG	NA	1209 (–/1182/27)	54
Sprague *et al.* ^24^	1993–2007	BRA-POR-TUR-VEN	NA	492 (–/485/7)	NA
Malluche *et al.* ^25^	2003–2008	USA-EUR	Caucasian-Black	630 (–/600/30)	0.6
Moore *et al.* ^26^	2005–2007	USA	Black	43 (–/43/–)	0
Jorgensen *et al.* ^27^	2012–2020	BEL	Caucasian	97	NA
Salam *et al.* ^28^	2013–2015	UK	NA	43 (28/15/–)	NA
Carbonara *et al.* ^5^	2015–2018	BRA	Caucasian-Black	260 (24/211/25)	25
Carbonara *et al.* ^6^	2015–2021	BRA	Caucasian-Black	386 (40/315/31)	36
Carbonara *et al.* ^29^	2015–2021	BRA	Caucasian-Black	275 (27/221/27)	35
Gentry *et al.* ^30^	2016	USA	NA	93 (–/83/10)	NA
Lavigne *et al.* ^31^	2016–2018	CAN	NA	11 (2/8/–)	NA
Aaltonen *et al.* ^32^	2016–2019	FIN	Caucasian	26 (–/26/–)	NA
Novel-Catin *et al.* ^33^	2020	FRA-BEL	NA	68 (–/–/–)	NA

As most studies did not provide information on the incidence of bone Al accumulation, in our opinion, the only way to know the true global prevalence of this condition in the current era is to systematically perform staining with solochrome azurine associated with staining with Pearls for iron detection in all bone samples from a representative sample of patients with CKD.

Until new evidence becomes available, we believe that nephrologists should consider bone Al accumulation as a possible diagnosis, even in asymptomatic patients with CKD under dialysis. An active search for Al detection in all bone biopsy samples from patients with CKD is advised. We must keep in mind that there is an established treatment for Al accumulation in bone^34^.

The standards for water quality in hemodialysis should have a “zero” Al limit as an ideal target. In addition, we propose a list of research gaps and key directions for research on this subject (Chart 1).

**Chart 1:** Proposed list information gaps and key directions for future research

Lack of or insufficient information	Proposed key direction
• Global prevalence of bone Al accumulation	Histological studies with active search for Al
• Serum Al detection sensitivity	Test random serum sample *vs*. post-desferrioxamine test
• Cutoff for Al concentration in water for HD	Test different cutoffs and their association with Al detection
• Frequency for measurements of Al in water	Test frequent random samples *vs*. semestral evaluation
• Al in raw materials for oral/IV drugs in dialysis	Detection of Al in these materials by mass spectrometry
• Al accumulation and cardiovascular risk	New epidemiological studies

## Conclusion

Our opinion is that bone Al accumulation should be considered as a potential frequent diagnosis, even nowadays. We urge other colleagues to actively search for this diagnosis in patients with CKD under dialysis in other geographic regions, including those who are presumably free of Al accumulation. Only then can we determine whether this is a restricted problem or a problem that chronically affects a larger population and with negative impact on clinical outcomes.

Meanwhile, efforts to improve the quality of water used for hemodialysis would be desirable, at least at local level, including reducing the allowed concentration of this metal in water to levels close to zero and increasing the frequency of measurements.
